# The Effect of Perioperative Music Listening on Patient Satisfaction, Anxiety, and Depression: A Quasiexperimental Study

**DOI:** 10.1155/2020/3761398

**Published:** 2020-02-07

**Authors:** Daryl Jian An Tan, Breanna A. Polascik, Hwei Min Kee, Amanda Chia Hui Lee, Rehena Sultana, Melanie Kwan, Karthik Raghunathan, Charles M. Belden, Ban Leong Sng

**Affiliations:** ^1^Department of Women's Anaesthesia, KK Women's and Children's Hospital 229899, Singapore; ^2^Duke University, Durham, North Carolina 27708, USA; ^3^Division of Nursing, KK Women's and Children's Hospital 229899, Singapore; ^4^Centre for Quantitative Medicine, Duke-NUS Medical School 169857, Singapore; ^5^Department of Music Therapy, KK Women's and Children's Hospital 229899, Singapore; ^6^Department of Anesthesiology, Duke University Healthcare System and Durham Veterans Affairs (VA) Healthcare System, Durham, North Carolina 27710, USA; ^7^Department of Health Policy and Management, University of North Carolina Gillings School of Public Health, Durham, North Carolina 27708, USA

## Abstract

**Background:**

The effect of perioperative music listening has been proven to relieve preoperative anxiety and depression, while improving patient satisfaction. However, music listening has not been extensively studied in Singapore. Therefore, the primary aim of our study is to investigate the patient satisfaction towards perioperative music listening in the local setting. The secondary aim is to investigate the effect of perioperative music listening in reducing patient surgery-related anxiety and depression.

**Methods:**

After obtaining ethics board approval, we conducted a quasiexperimental study on a cohort of female patients who were undergoing elective minor gynaecological surgeries. Apple iPod Touch™ devices containing playlists of selected music genres and noise-cancelling earphones were given to patients to listen during the preoperative and postoperative periods. Hospital Anxiety and Depression Scale (HADS), EQ-5D-3L questionnaire, music listening preferences, and patient satisfaction surveys were administered. Wilcoxon signed-rank and McNemar's tests for paired data were used for analysis.

**Results:**

83 patients were analysed with 97.6% of patients in the preoperative period and 98.8% of patients in the postoperative period were satisfied with music listening. The median (IQR [range]) score for preintervention HADS anxiety was 7.0 (6.0 [0–17]), significantly higher than that in postintervention at 2.0 (4.0 [0–12]) (*P* < 0.001). Similarly, there was a significant reduction in preintervention HADS depression as compared to postintervention (*P* < 0.001). Similarly, there was a significant reduction in preintervention HADS depression as compared to postintervention (

**Conclusion:**

Perioperative music listening improved patient satisfaction and can reduce patient anxiety and depression. We hope to further investigate on how wider implementation of perioperative music listening could improve patient care.

## 1. Introduction

The use of music listening in the perioperative setting has been validated in numerous studies [1]. Compared to the conventional use of pharmacological drugs to reduce postoperative pain and anxiety, music listening has been shown to be safer and more cost-effective [2], reduce perioperative pain [3], and improve overall patient satisfaction [4, 5]. Additionally, music listening can modulate the patient's inflammatory response and reduce perioperative anxiety [6–8].

In Singapore, the implementation of music listening as a noninvasive intervention in the perioperative setting is limited. Given the potential clinical benefits of music listening as justified by the aforementioned studies, the primary aim is to investigate the patient satisfaction towards perioperative music listening in the local setting. The secondary aim of our study is to investigate the effect of perioperative music listening in reducing patient surgery-related anxiety and depression in the local setting. The instruments used to address the secondary aim include the Hospital Anxiety and Depression Scale (HADS) and EQ-5D-3L questionnaires. The HADS comprises fourteen questions with individual scores from 0 to 3, to produce a final score for the assessment of anxiety and depression on a continuum scale. Similarly, the EQ-5D-3L assesses the general health status through questions exploring five dimensions: mobility, self-care, usual activities, pain/discomfort, and anxiety/depression.

## 2. Materials and Methods

After obtaining approval from Singhealth Centralized Institutional Review Board (CIRB Ref: 2017/2292) and trial registration from clinicaltrials.gov (Ref: NCT03226028), we conducted a quasiexperimental study on a cohort of patients to investigate the effect of perioperative music listening in KK Women's and Children's Hospital, Singapore. This study adhered to the CONSORT guidelines. We recruited female patients who were not hearing impaired, of American Society of Anesthesiologists (ASA) physical statuses 1 and 2, and were undergoing elective minor gynaecological surgeries, mainly dilation and curettage procedures and hysteroscopies. Obstetric patients and patients who were unable to read and understand the questionnaires of this study were excluded from this study.

Upon obtaining written informed consent, patients' demographic data and music listening preferences were collected. The Hospital Anxiety and Depression Scale (HADS) and EQ-5D-3L questionnaire were administered to the patients to determine the patient's baseline anxiety and depression levels. Thereafter, the patients were given an iPod Touch™ device (Apple; Cupertino, CA, USA) along with noise cancellation earphones, which contained 1,441 songs across 34 presaved playlists spanning different music genres. These playlists were compiled by the institution's music therapist to ensure adequate coverage of the various music genres. Patients were able to listen to any playlist of their choice during the preoperative period in a quiet environment until their elective surgeries. Thirty minutes prior to their scheduled surgeries, all patients were asked to listen to a mandatory music playlist, compiled by the institution's music therapist, which promoted relaxation and serenity. The preoperative music listening was discontinued when the patient entered the operating theatre for her scheduled surgery. The duration of music listening, patient's preferred music playlists, and patient satisfaction were collated. The time between when the patient stopped listening to music and the start of anesthesia administration was also noted.

Intraoperative vital signs, analgesic usage, and duration of surgery were recorded. All patients received routine anesthesia and surgical protocols and were sent to the recovery unit after surgery. Patients resumed listening to music once they were ready and comfortable to do so and would continue until their discharge from the recovery unit. Thereafter, patients were administered a second set of HADS and EQ-5D-3L questionnaire and interviewed on their satisfaction, preferred music playlists, and their overall experience with the music listening study. Postoperative vital signs, analgesic usage, pain scores, and duration of stay in the recovery unit were also recorded. The iPod Touch™ devices and noise-cancelling earphones were disinfected in accordance with the institution's infection control guidelines, replacing the earbuds with fresh ones.

Patient satisfaction was assessed by asking patients to rate their satisfaction with their overall experience using a 4-point verbal rating scale (1 = excellent, 2 = good, 3 = fair, and 4 = poor). In assessing anxiety and depression, the HADS and EQ-5D-3L self-reported questionnaires were administered. Out of the five dimensions in the EQ-5D-3L questionnaire exploring questions relating to mobility, self-care, usual activities, pain/discomfort, and anxiety/depression, only pain/discomfort and anxiety/depression dimensions were analysed as the former three dimensions would be confounded by the surgery itself. Similarly, the visual analog scale examining the patient's self-rated health in the EQ-5D-3L questionnaire was not applicable and therefore excluded from this study.

### 2.1. Statistical Analysis

Statistical analysis was performed using SAS version 9.3 software (SAS Institute; Cary, NC, USA). The collated data were summarized as mean and standard deviation (SD) for normally distributed numerical data, median, and interquartile range (IQR) for nonnormally distributed numerical data, and frequency and proportion for categorical data. Normality assessments were performed, and the results of this study do not follow a normal distribution. Wilcoxon signed-rank test and McNemar's test for paired data were used for analysis, and significance was taken to be *P* < 0.05.

## 3. Results

**Figure 1:**
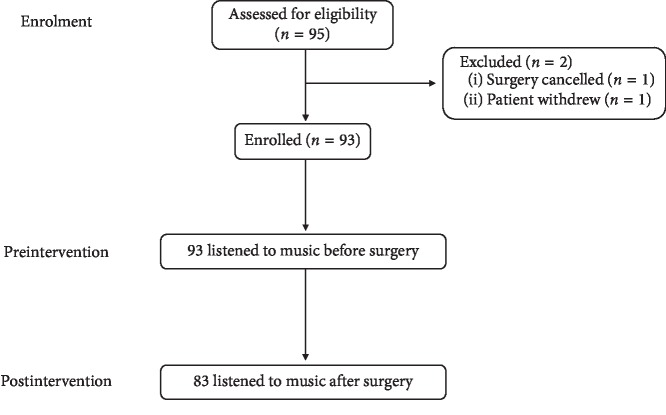
Methodology of the study.

Ninety-five patients were assessed for eligibility for the study, but only 83 of them were included in the study data analysis. Two patients were excluded from the study: one patient had surgery postponed and another patient did not wish to continue with the study due to abdominal pain. After surgery, 12 patients declined to listen to music, with 7 (58.3%) patients reported drowsiness and 4 (33.3%) patients reported pain being the most common reasons for refusal. These were excluded from the data analysis to preserve paired data analysis. The flowchart of the study is represented in Figure 1.

Table 1 reflects the baseline and demographic data of the recruited patients. The mean ± SD duration of music listening in the preoperative period was 128.6 ± 60.88 minutes and 43.4 ± 19.41 minutes in the postoperative period. The mean ± SD duration between the cessation of music listening in the preoperative period to the induction of general anesthesia was 5.1 ± 2.33 minutes.  Table 2 represents the results from the questionnaires obtained from patients before the initiation of music listening and after the conclusion of the study. In terms of patient satisfaction, 81 (97.6%) patients in the preoperative period and 82 (98.8%) patients in the postoperative period were satisfied (rated “*excellent*” and “*good*”) with music listening.

**Figure 2:**
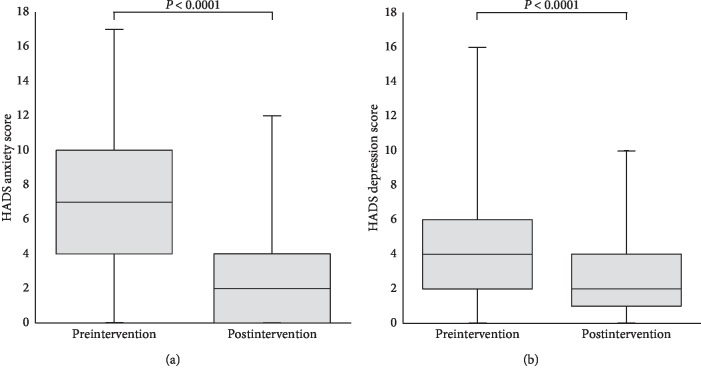
Box-and-whisker plots showing (a) HADS anxiety scores and (b) HADS depression scores in both preintervention and postintervention periods. Horizontal lines are median, boxes are IQR, and whiskers are range.

The median (IQR [range]) score for preintervention HADS anxiety was 7.0 (6.0 [0–17]), significantly higher than that in postintervention at 2.0 (4.0 [0–12]) (*P* < 0.001). Similarly, there was a significant reduction in preintervention HADS depression as compared to postintervention (*P* < 0.001).  Figure 2 illustrates the box-and-whisker plots for both HADS anxiety and depression scores. These results were corroborated by similar findings from the EQ-5D-3L anxiety/depression dimension (*P*=0.003). 94.0% of patients in the preoperative period reported experiencing no pain at rest as compared to 79.5% of patients in the postoperative period (*P*=0.007).

Table 3 represents the patient preferences of song genres in the preoperative and postoperative periods. The ambient genre (22.9%) was most favoured in the preoperative period, followed by modern (19.3%) and religious (18.1%) genres. However, the mandatory playlist prior to surgery (19.3%), ambient (16.9%), and religious (14.5%) genres were most popular amongst patients in the postoperative period.

## 4. Discussion

This study examined the efficacy of perioperative music listening in reducing patient surgery-related anxiety and depression in the local setting. Majority of patients participated in both preoperative and postoperative music listening. Patients preferred to have their own choice of music, although a recommended mandatory playlist was offered. There is a wide spectrum of music genres chosen by Asian patients in this study. Majority of patients in both preoperative and postoperative periods were satisfied with perioperative music listening. We found that patients who listened to music in the perioperative setting reported feeling less anxiety and depression scores.

The therapeutic benefits of music have been well expounded in the current research literature. It is widely proposed that listening to music helps to distract patients from focusing on negative thoughts of the impending surgery, a major source of anxiety [9, 10]. Listening to music provides a source of serenity and calmness that patients can divert their attention to, and focus less on feelings of anxiety and fear [11]. However, the influence of perioperative music listening has not been far-reaching in Singapore, with only one other study investigating the effect of music listening on postoperative pain and anxiety in orthopaedic surgical patients [12]. We hope that our study can generate more interest in considering perioperative music listening as a possible nonpharmacological approach to alleviating psychological distress in patients undergoing surgery in the local and regional setting.

From our study, perioperative music listening significantly reduces psychological distress of anxiety and depression in patients. Our findings are consistent with those made in previous studies [7, 8, 11, 13]. To many people, surgery is a daunting experience that comes with emotional vulnerabilities [14]. These emotions are often intensified moments before surgery causing overwhelming anxiety and even depressive moods. Preoperative anxiety and depression has extensive consequences if left unattended. Increased preoperative anxiety can lead to postponement or even cancellation of planned surgeries, and increase in dose requirements of anesthetic drugs to ensure unconsciousness intraoperatively prolonged hospital stay and poorer overall patient satisfaction [15, 16]. Similarly, preoperative depression has been shown to increase the risk of postoperative delirium in selected patient groups [17, 18]. Therefore, there is great justification in addressing preoperative psychological distress. Music listening is affordable and safe and can be easily used by healthcare professionals in the hospital [19]. It counteracts psychological distress efficiently with a very low risk profile.

We acknowledge several limitations of our study. As most of our findings were gathered from questionnaires, our results could have been affected by patients' willingness to take part in this study protocol and this could be different during routine clinical practice. Moreover, the ability to read and understand English and Chinese in the questionnaires resulted in a selected patient population. We also did not investigate factors that may have affected patient's anxiety and mood such as interactions between the study team investigator and patient, as well as the responsiveness to patients' needs. Lastly, postintervention results may have been confounded by the patient's knowledge that the surgery itself, the main source of anxiety, had been successful. Nevertheless, it is noteworthy that the efficacy of perioperative music listening is well established from studies conducted abroad, as a safe, inexpensive, and effective option. We hope that our study can provide fresh perspectives into the use of perioperative music listening for this purpose in the local and regional perioperative setting.

## 5. Conclusion

In conclusion, the findings of our study are in congruence with those made in previous studies that perioperative music listening is an effective approach in reducing psychological distress. We believe that this study outlining our experiences with the implementation of perioperative music listening in Singapore can be used for future research in the local healthcare settings, further improving patient outcomes with lower cost, greater convenience, and safety.

## Figures and Tables

**Table 1:** Patient demographics.

Characteristics	*N* = 83
Age (years)	38.9 ± 8.66
Race	
Chinese	62 (74.7%)
Malay	15 (18.1%)
Indian	3 (3.6%)
Others	3 (3.6%)
ASA status	
Class 1	66 (79.5%)
Class 2	17 (20.5%)
Duration of music listening (min)	
Preoperative period	128.6 ± 60.88
Postoperative period	43.4 ± 19.41
Duration of surgery (min)	24.0 ± 15.90
Duration of stay in the recovery unit (min)	63.3 ± 23.70
Incidence of patients using opioids for analgesia in the recovery unit	10 (12.0%)

Values are represented as mean ± SD or number (proportion). ASA: American Society of Anesthesiologists.

**Table 2:** Preintervention and postintervention psychological outcomes.

Variables	Preintervention	Postintervention	*P* value
HADS score			
Depression	4.0 (4.0 [0–16])	2.0 (3.0 [0–10])	<0.001
Anxiety	7.0 (6.0 [0–17])	2.0 (4.0 [0–12])	<0.001
EQ-5D-3L dimensions anxiety/depression			0.003
Not anxious/depressed	54 (65.1%)	83 (100.0%)	
Having anxious/depressed	29 (34.9%)	0	
Pain/Discomfort			0.007
No pain/discomfort	78 (94.0%)	66 (79.5%)	
Having pain/discomfort	5 (6.0%)	17 (20.5%)	

Values are represented as median (IQR [range]) or number (proportion). HADS: Hospital Anxiety and Depression Scale; EQ-5D-3L: three-level version of the EuroQol five-dimensional questionnaire.

**Table 3:** Collation of preselected music genre preferences.

Music genres	Preoperative	Postoperative
Mandatory playlist prior to surgery	9 (10.8%)	16 (19.3%)
Chinese songs	14 (16.9%)	8 (9.6%)
Religious themed	15 (18.1%)	12 (14.5%)
Disney tunes	12 (14.5%)	7 (8.4%)
Instrumental	14 (16.9%)	7 (8.4%)
Modern	16 (19.3%)	6 (7.2%)
Oldies	10 (12.0%)	6 (7.2%)
Ambient	19 (22.9%)	14 (16.9%)
Indian songs	2 (2.4%)	2 (2.4%)
Korean songs	2 (2.4%)	1 (1.2%)
Malay songs	1 (1.2%)	1 (1.2%)
Movies and musicals	3 (3.6%)	1 (1.2%)
Waltz and tango	0 (0.0%)	1 (1.2%)

Values are represented as number (proportion).

## Data Availability

All data generated or analysed during this study are included in this manuscript. The data used to support the findings of this study are available from the corresponding author upon request.

## Abbreviations

ASA: American Society of Anesthesiologists
CIRB: Centralized Institutional Review Board
EQ-5D-3L: Three-level version of the EuroQol five-dimensional questionnaire
HADS: Hospital Anxiety and Depression Scale
SD: Standard deviation
IQR: Interquartile range.
